# Cystic Periventricular Leukomalacia: A Condition that Became Uncommon in the Premature Neonate, Diagnosed on Transcranial Ultrasound

**DOI:** 10.5334/jbr-btr.1000

**Published:** 2016-01-29

**Authors:** Laurent Van Camp, Luc Steyaert

**Affiliations:** 1 AZ Sint-Jan Brugge, BE

**Keywords:** Neonate, transcranial ultrasound, periventricular leukomalacia, pediatrics

## Abstract

A preterm neonate was born in our center, as a part of a diamniotic dichorionic twin pregnancy, complicated with placental abruption with need for urgent cesarean section at 26 weeks of gestation. After a difficult neonatal start with respiratory and cardiovascular problems, the neonate safely reached the neonatal intensive care unit. Further work-up and supportive care was continued. Transcranial ultrasound imaging through the anterior fontanel was part of this work-up. Initial examinations were not normal, and showed cystic lesions along the germinal matrix, without hydrocephalus or parenchymal lesions. The findings were highly suggestive of sequellae of grade II germinal matrix bleed according to Papile and Burstein (Image A). Further follow-up examinations by means of ultrasound depicted a discrete enlargement of the lateral ventricles over time (Image B). Later on development of widespread periventricular cystic lesions became apparent (Image C and D). The cystic lesions are located in the periventricular white matter, and are not attached to the ventricles. This finding makes cystic periventricular leukomalacia the preferred diagnosis, and makes venous infarction with cystic alterations secondary to germinal matrix bleeding less likely.

A preterm neonate was born, as a part of a diamniotic dichorionic twin pregnancy, complicated with placental abruption with need for urgent cesarean section at 26 weeks of gestation. The neonate was transferred to the neonatal intensive care unit after attending to his initial respiratory and cardiovascular issues. Further work-up and supportive care was continued.

Transcranial ultrasound imaging through the anterior fontanel was performed on day two. The examination showed cystic lesions along the germinal matrix, without hydrocephalus or parenchymal lesions. The findings were highly suggestive of sequellae of grade II germinal matrix bleed according to Papile and Burstein (Figure 1).

**Figure 1:**
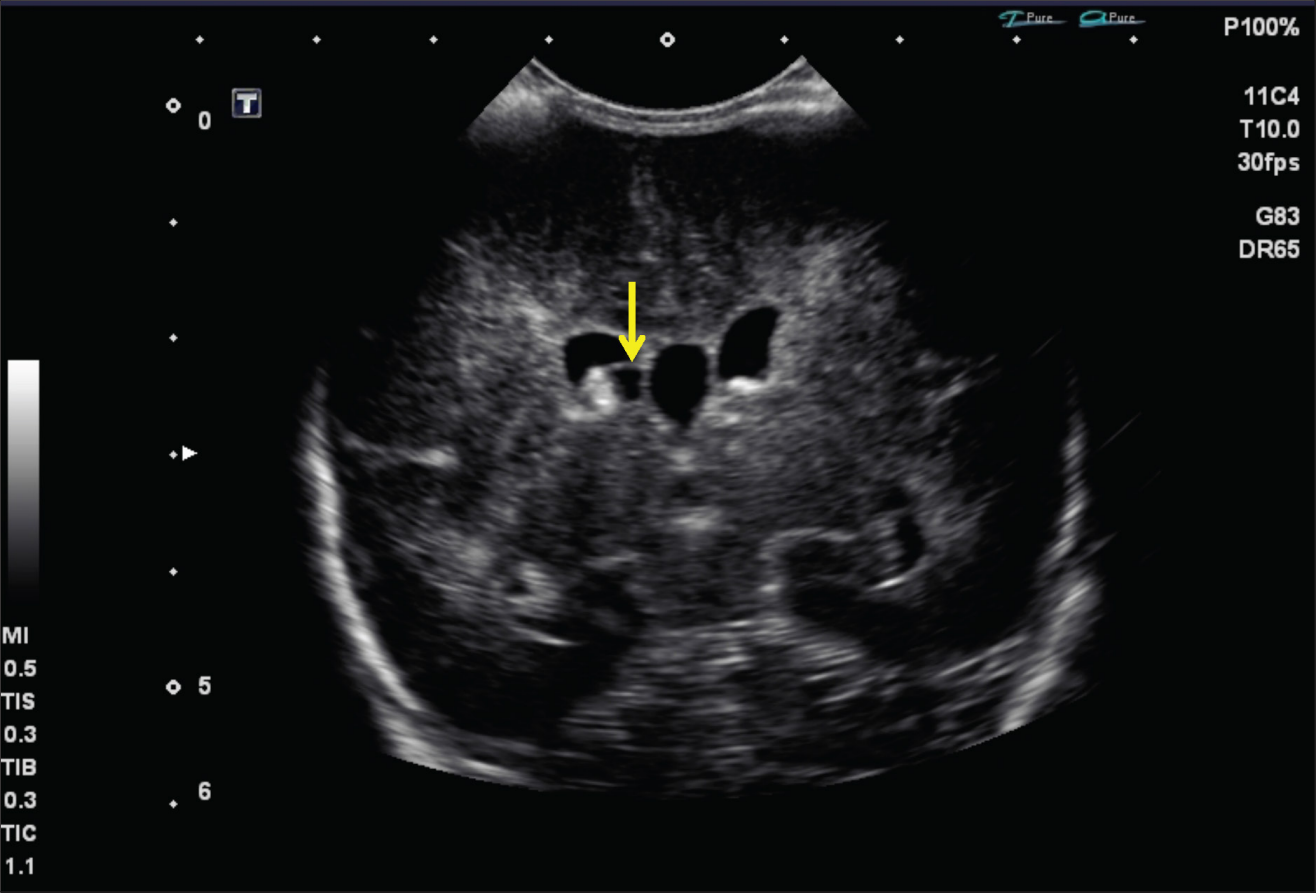
Transcranial ultrasound, day 2, coronal plane. Cystic lesion in proximity of the germinal matrix (arrow), no hydrocephalus.

Weekly follow-up examinations by means of ultrasound depicted a discrete enlargement of the lateral ventricles first seen two weeks post-partum (Figure 2).

**Figure 2:**
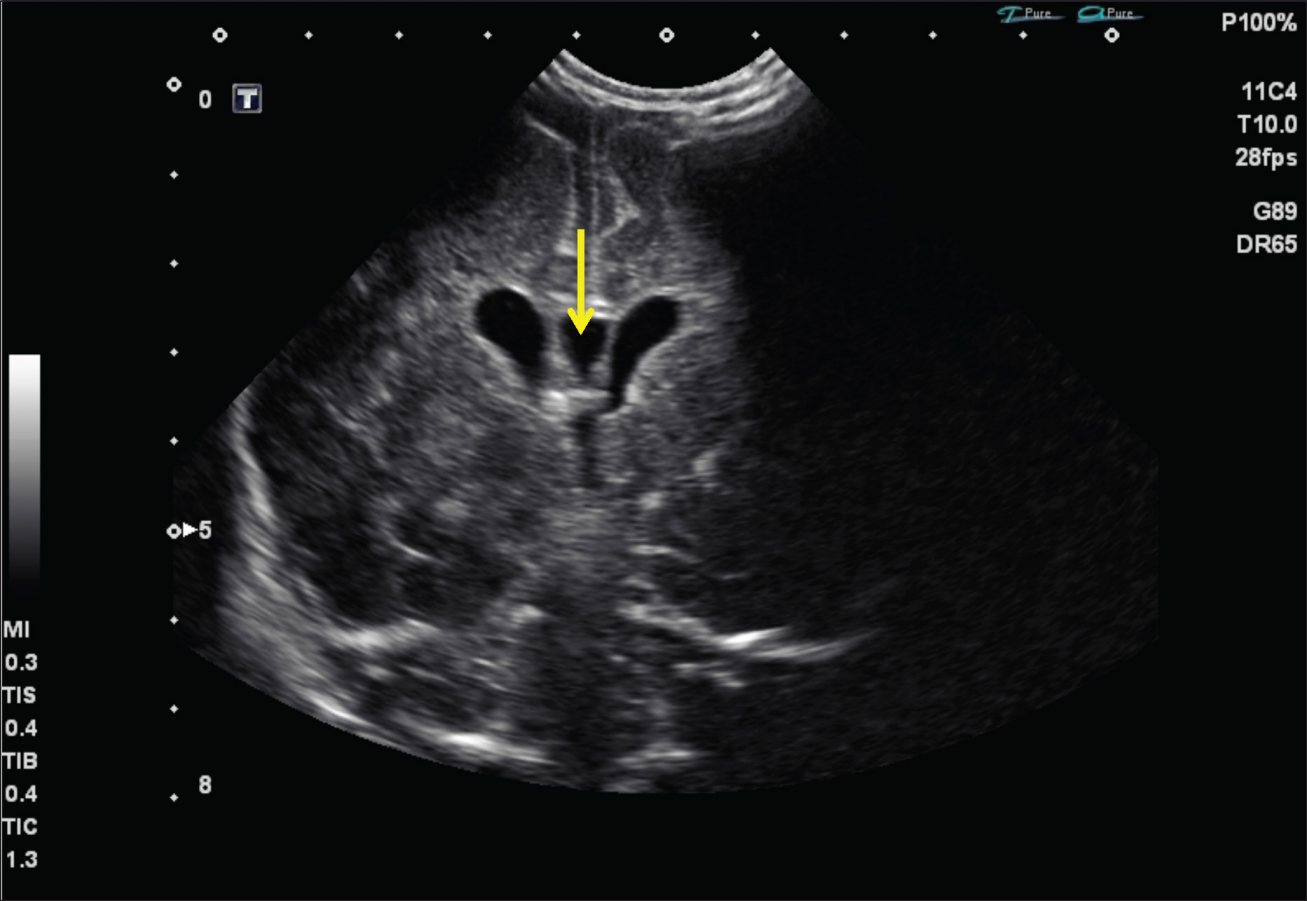
Transcranial ultrasound, day 16, coronal plane. Discrete enlargement of the third ventricle and lateral ventricles. Persistent cavum septum pellucidum, normal variant (arrow).

At 6 weeks old, some periventricular cystic lesions became apparent, and extensive periventricular cysts could be readily diagnosed at week 8 post-partum (Figures 3 and 4). The cystic lesions are located in the periventricular white matter, and are not attached to the ventricles. Cystic periventricular leukomalacia was the preferred diagnosis in this patient.

**Figure 3:**
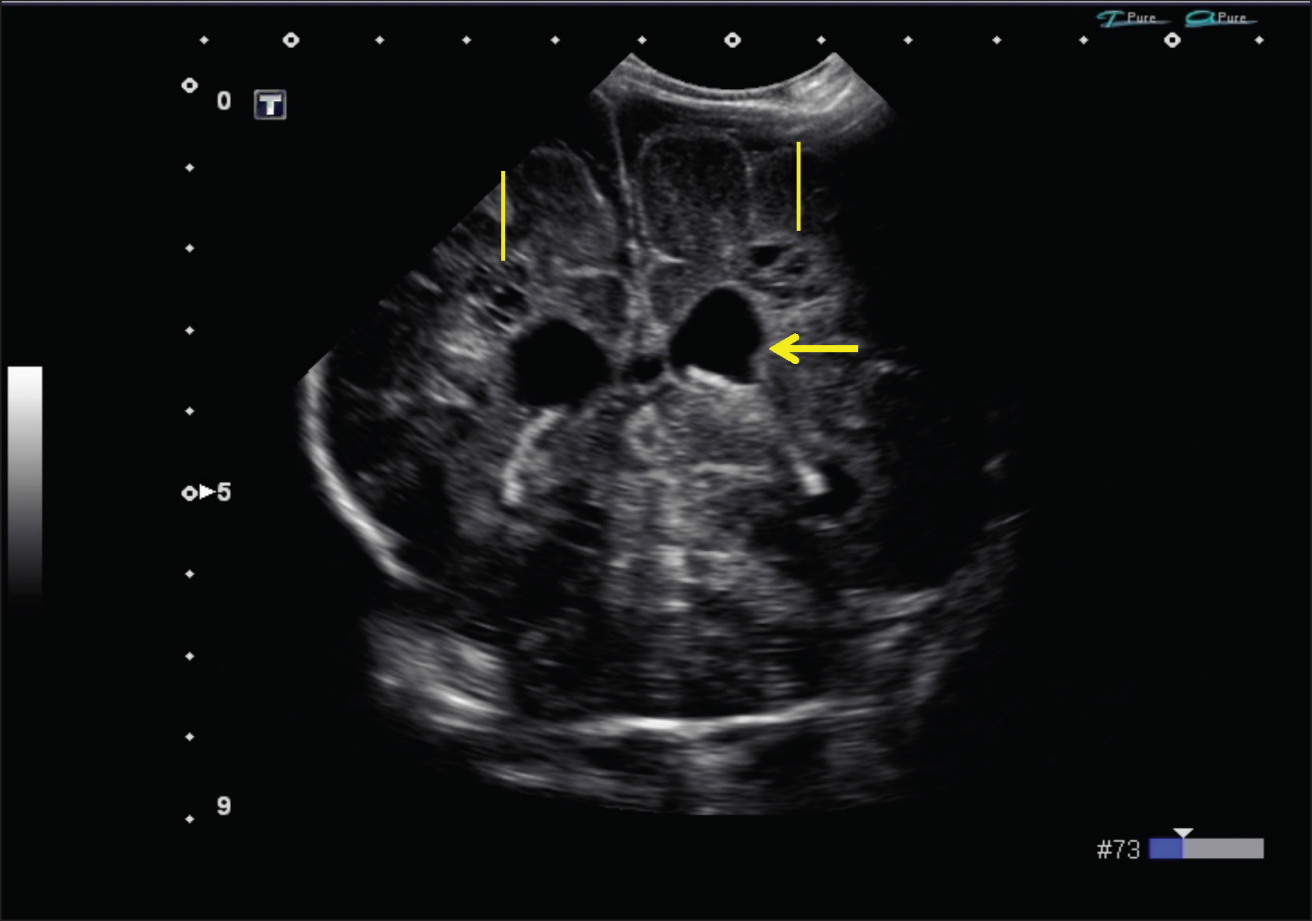
Transcranial ultrasound, 8 weeks post-partum, coronal plane. Progressive enlargement of lateral ventricles (arrow), and formation of periventricular cysts (straight lines).

**Figure 4:**
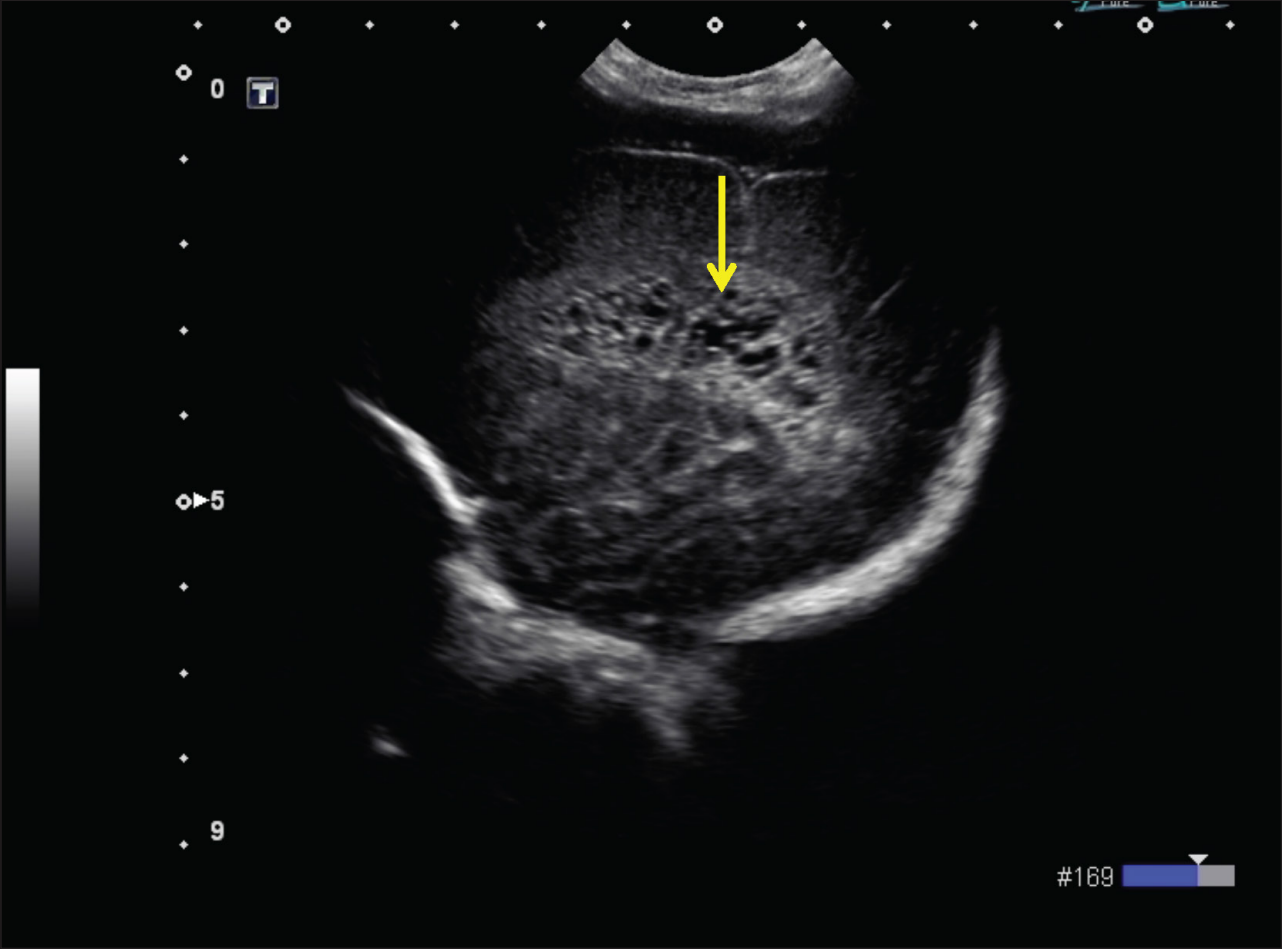
Transcranial ultrasound, 8 weeks post-partum, parasagittal plane. Extensive periventricular cysts (arrow).

## Discussion

The premature neonate and its brain are still in a developmental stage, and are more prone to perinatal stress and accompanying cerebral hypoperfusion injury than the term child [1,2,3]. In contrast to the latter, the vulnerability of the brain in the neonate to the aforementioned injuries is located in the periventicular white matter, as a consequence of incomplete maturation of the vasculature [2]. Periventricular leukomalacia (PVL) still remains one of the most frequently diagnosed types of brain damage in this population [3], although the sizeable improvements in neonatal intensive care medicine now allow premature infants a higher chance of survival without such injuries. It is important to diagnose and indicate this type of damage to the clinician, because it can indicate important neurological morbidities over the years to come, may suggest further diagnostic work-up, with electroencephalogram and MR-imaging, or in rare cases have impact on treatment and prognosis [2].

Transcranial ultrasound in the neonate is a convenient, radiation-free, straightforward and cheap imaging modality fit for bedside diagnosis and follow-up tool for cerebral pathology early on in life. Screening for, and depicting the severity of white matter lesions, intracranial hemorrhage and hydrocephalus are the primary incentives for performing routine transcranial ultrasound scanning in the preterm neonate [2]. As such, ultrasound is the mainstay diagnostic tool for PVL in the neonate [3,4]. Periventricular white matter alterations may become apparent as soon as within 24 to 48 hours, and evolve over time, indicating the need for repeated examinations [4]. Transcranial ultrasonography in the neonate allows for adequate diagnosis of PVL, with a proposed sonographic system according to De Vries et al [5]. Cystic periventricular leukomalacia presents a severe lesion, grade 3 or 4, depending on the extent of the lesion [4,5]. Cystic PVL is seen more and more rarely in present time because of the important steps neonatal intensive care has taken. Though it is a seldom diagnosis, the sonographic manifestation of cystic PVL is important to detect, because of the increased risk of developing extensive neurological comorbidities and specifically cerebral palsy, with worse outcome in later life.

Disadvantages of ultrasound are its lower sensitivity compared to MR, the observer variability, and the differentiation of low-grade lesions [4]. Alternatives such as MR-imaging are present, but carry the inherent strain of needing general anesthesia for adequate image acquisition, and present some operational difficulties. Nevertheless, it should be noted that MR-imaging is the most sensitive and specific imaging tool for hypoxic or ischemic brain injury in the infant [2]. CT of the brain is rarely used in this population.

From a diagnostic point of view, a combination of serial ultrasound examinations and single MR-imaging in the infant with higher grade PVL seems appropriate [4].

In summary, this case of a rather rare finding of cystic PVL should refocus our interest in an important neonatal imaging technique. The importance of transcranial ultrasonography in the multimodality imaging in this specific population cannot be overstated because of the important information it can reveal, the practical advantages it carries and the evolution over time it can easily exhibit. Though the treatment potential of the findings may seem limited at first, the radiologist can provide substantial diagnostic and prognostic information that will facilitate the overall management of these young children through time.

## Competing Interests

The authors declare that they have no competing interests.
